# Supplementary material for: Improved synapsis dynamics accompany meiotic stability in Arabidopsis arenosa autotetraploids
Source: Proc Natl Acad Sci U S A. 2025 May 7;122(19):e2420115122. doi: 10.1073/pnas.2420115122 (PMC12088413; doi:10.1073/pnas.2420115122)
Supplement: Supplementary file 1 — Appendix 01 (PDF) [file pnas.2420115122.sapp.pdf]

Supporting Information for

Changes in synapsis dynamics accompany variation in meiotic stability in *Arabidopsis arenosa* autotetraploids

Authors: Adrián Gonzalo[1]*, Aditya Nayak[1], Kirsten Bomblies[1]*
1. Institute of Molecular Plant Biology, Department of Biology, ETH Zürich, Zürich, Switzerland.
*Correspondence to Kirsten Bomblies and Adrian Gonzalo
Email: kirsten.bomblies@biol.ethz.ch and adrian.gonzalo@biol.ethz.ch

This PDF file includes:

Supporting text
Figures S1 to S15
Table S1
SI References

Other supporting materials for this manuscript include the following:

Datasets S1 to S7

Supporting Information Text

General procedure for SIM image acquisition and visual inspection: Since fluorescence intensities often show experimental variation between cells, images were acquired on autoscale mode, which adjusts the exposure to the brightest element in the field. Consequently, for visual comparison of images, it is recommended to eliminate negative intensities, by setting the "minimum displayed value" to 0, in the brightness and contrast settings, so the lowest intensity pixels are displayed as black.

General procedure for automated analysis of SIM images. Each SIM image stack was first inspected. Stacks with images having too much background signal in one of the channels were discarded. Moreover, image stacks that lacked one of the channels were also discarded since our Fiji macros can only work on images with four channels. The macros are designed to be applied in files with channels ordered as follows: DAPI, ASY1, ZYP1, HEI10. Files with a different order of channels need to be reordered. This can be done by adding one initial step to the existing macro using the "Arrange channels" command. Moreover, these macros require that the images contain scale information in their metadata that can be accessible by Fiji. All macros are available in https://github.com/adgon/MeioScope.

Each macro processes the signal from one or more channels in two steps. The first step is signal detection and the criterion of what is detected as signal and what is not is determined by different preestablished thresholding methods, namely, Yen (72), Moments (73) and default (ordered from less to more sensitivity). The second step is the measurements on the detected signals which can be signal count, intensity measurement, or length measurements.

Thresholding methods are algorithms used to separate foreground signals (e.g., HEI10 foci) from the background in an image by determining the minimum intensity required for a pixel to be classified as foreground. We considered as prominent HEI10 foci those that passed the Yen threshold, while total HEI10 signals were defined as those that passed the Moments threshold. Intensities below the Moments threshold were classified as background. For ZYP1 signal, we used the default thresholding method. We selected the Yen and Moments methods from the available options in Fiji because they consistently matched our visual assessment of the images. All thresholding methods analyze the frequency histogram (i.e. the distribution) of pixel intensities in the image. The Yen method finds the threshold that maximizes the total entropy (a measure of information content) of the image, effectively separating foreground and background based on the distribution of pixel intensities. In contrast, the Moments method finds the threshold that best preserves the statistical properties (or moments, namely; mean, variance, skewness, and kurtosis) of the original image in the thresholded result. Because thresholding is based on relative pixel intensities, the same method may produce different cutoff values for different images (see examples in Fig. S13). After thresholding, we measured the detected signals, including signal count, intensity, and length.

Macro 1 analyzes HEI10 channel and provides two different output tables. In the first of them, with the count data of prominent foci (detected with Yen) that totally or partially overlap with synapsed regions (ZYP1 signal, detected with default). The second output provides the HEI10 intensities from both prominent foci (detected with Yen) and the total signal (detected with Moments). The percentage of intensity from prominent foci relative to the total signal was later used to calculate the HEI10 accumulation level. For this analysis, each and every cell was processed with automatic thresholding, allowing no exception for manual thresholding to prevent any bias. The workflow of this macro is illustrated in Fig. S14.

Macro 2 measures the total 3D lenth of ASY1 whose signal was thresholded using Yen method. This measurement provides a measure of the extent of asynapsis. In the vast majority of cells, the threshold detects ASY1 highly specifically at asynaptic regions where the axis is not remodeled

(79) and intense ASY1 signals persist, while the dim signal at remodeled axes after synapsis are not detected (Fig. S2F). However, in some rare cells, manual thresholding was required (Fig. S15). Macro 3 (optional), quantifies 2D length of ASY1 and ZYP1 linear signals (thresholded with the Yen and default methods, respectively). These data were not analyzed in this work, but they can be useful for quality control purposes, to identify cells where manual thresholding is required. For instance, rare cells with an excessive length of both ASY1 and ZYP1 correspond to cases where ASY1 was overestimated (Fig. S15). Conversely, cases where ASY1 length in 2D is greater than in 3D, indicates an underestimation. Finally, visual inspection ASY1 thresholdded signal in Fiji can also be used for total certainty.

General procedure for statistical modeling. Generalized Linear Mixed Models (GLMMs) are used to predict the behavior of a response variable (e.g., ASY1 length or univalent count) as a function of one or more explanatory variables. Explanatory variables of primary interest, which are assumed to be measured without error, are referred to as fixed effects (e.g., HEI10 accumulation level or genotype). Explanatory variables that are not of primary interest but need to be controlled for, and are assumed to be drawn from a larger population, are referred to as random effects (e.g., individual or scorability class). The formulas for both fixed and random effects are specified in Supporting Dataset S7. In our models, we considered either a single random effect (e.g. plant individual) or a nested random effect (e.g., individual nested in genotype). Random effects models with more than two variables, or complex nested structures, were too complex given the amount of data available. When random effects were not included, we used Generalized Linear Models (GLMs) instead. To predict the behavior of a response variable using GLMs or GLMMS, it is necessary to specify the distribution family that reflects the nature of the response variable. For continuous variables (such as ASY1 length) we used Gamma or Gaussian distributions, whereas for discrete count data (such as multivalent counts), we used Poisson or negative binomial distributions. The negative binomial distribution was selected for count data when overdispersion (i.e., variance greater than the mean, a property of Poisson) was observed, as it accounts for the extra variability in the data. Additionally, generalized models require a link function, which transforms the linear combination of explanatory variables to the scale of the response variable and determines the shape of the predicted curve. For example, the log link function confers an exponential relationship between the predictors and the response. In cases where the data contained an excess of zero values, we included a zero-inflation term to appropriately handle the overabundance of zeros in the data set.

In each analysis, several models were fitted using different formulas (varying random effects, distributions, link functions, etc.; see Supporting Dataset S7), with the glmmTMB (75) R package used for all cases except for negative binomial-GLMs, which were fitted using the MASS R package. Models that failed to fit the data when using glmmTMB were discarded. Next, we used the Performance R package (76) to compare model metrics. Specifically, we compared the coefficients of determination (R^2) across models. For GLMMs, two types of R^2 were calculated: the conditional R^2, which accounts for both fixed and random effects, and the marginal R^2, which considers only fixed effects. In linear models, R^2 indicates the proportion of observed variability that can be explained by the model (larger R^2 suggesting a better fit) It should be noticed, however, that the interpretation of the R^2 is more complex for GLM(M)s, although this metric is still used as it has similar properties in terms of explanatory power. (see (76) for detailed interpretation in the context of GLM/GLMMs). Additionally, we evaluated models using Akaike's Information Criterion (AIC) and Bayesian Information Criterion (BIC), which measure the balance between model accuracy and complexity. Lower AIC and BIC values indicate better-performing models, with these criteria helping to penalize overfitting while still prioritizing explanatory power. Based on these metrics (detailed for each model fitted in Supporting Dataset S7), we selected the most promising models for each analysis for further residuals-based model diagnostic checks.

For residuals-based model diagnostics, we used the DHARMa R package (77). First, we verified that the variables included in the model did not exhibit collinearity. Additionally, we ensured that

the model was not “singular”, which is a strong indication of either collinearity or an overly complex and overfitted model (77). Models detected as singular were discarded due to their potential to have near-zero power. Next, we used the residuals to test four key assumptions: normality of residuals, absence of over- or under-dispersion, no excess of outliers, and homogeneity of variance (detailed descriptions of these tests are provided in Supporting Dataset S7). For testing variance homogeneity, DHARMa conducts multiple tests and generates diagnostic plots of the residuals. In cases where these plots showed no clear patterns and variance was roughly homogeneous across groups (e.g. genotypes), we tolerated minor heteroskedasticity (i.e., variance heterogeneity of the residuals). To select the best fitting model for each analysis, we prioritized models that had the best Performance metrics (R^2, AIC, and BIC) and successfully passed the DHARMa diagnostics. Further details for each analysis are provided below.

Modeling multivalent count in Metaphase I. We tested different random effect structures, considering “genotype”, “plant”, and “scorability” classes as random effects. As explained in the Data Generation and Analysis section, the scorability class reflects the level of confidence in scoring each cell. However, because models including all three random effects failed the singularity test, we proceeded with models containing only one or two random effects terms. Models with a single random effect term performed better, with “scorability” class outperforming individual and nested structures involving both terms. Additionally, models that included only cells from the best scorability classes (A and B) performed better than those that incorporated all cell classes. Therefore, for further analysis, we compared models that used only these high-quality cells.

Modeling univalent count in Metaphase I. The modelling of univalent count data followed a similar approach as described for multivalent count (see above), with a difference that this time, a nested structure of random effects (involving both “scorability” class and “plant” individual) provided a better performance.

Modeling asynapsis decay with HEI10 accumulation. Minor heteroskedasticity (heterogeneous variance) was an issue in all the models tested, but the best fitting model did not show any discernible pattern in the residuals plot. Notably, variance was homogeneous within and among genotypes.

Modeling late asynapsis decay with HEI10 accumulation. For this analysis, there were no significant issues to report.

Modeling the effect of colchicine on NEO-4X behavior. For consistency, we used the same model formula applied to the analysis of asynapsis decay with HEI10 accumulation. No additional models were evaluated. The selected model had minor heteroskedasticity, though no obvious patterns were present in the residual plots.

Modeling dynamics of synapsis initiation. All models that included random effects were singular, indicating that the model complexity exceeded what the data could support. Therefore, only GLMs were tested. Although count data typically requires discrete distributions, Gaussian models showed reasonable performance, which warranted testing. However, the best-fitting model was ultimately based on count-data distributions (Poisson).

Modeling dynamics of early synapsis elongation. This analysis exhibited the same issues as those encountered during the modelling of synapsis initiation.

Modeling crossover increase with asynapsis. Applying a log transformation to the explanatory variable substantially improved the models, suggesting that the relationship was logarithmicrather than linear. In addition, GLMs performed better than GLMMs which, in all cases, were singular and with lower explanatory power.

Modeling the effect of extensive asynapsis on crossover number. Most models tested showed signs of overfitting (singularity, but not collinearity). The best fitting model without singularity did not include random effects and was fit using GLM. While minor heteroskedasticity was present, the variance was homogeneous across both plants and genotypes.

Modeling decay of asynaptic parallels with ASY1 length. For this analysis, there were no significant issues to report.

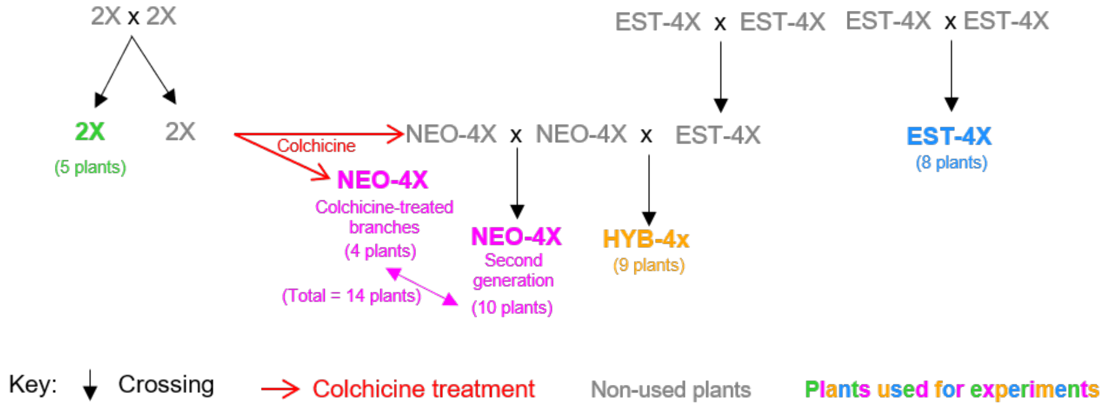

Fig. S1. Scheme of plant materials generated and used in this study.

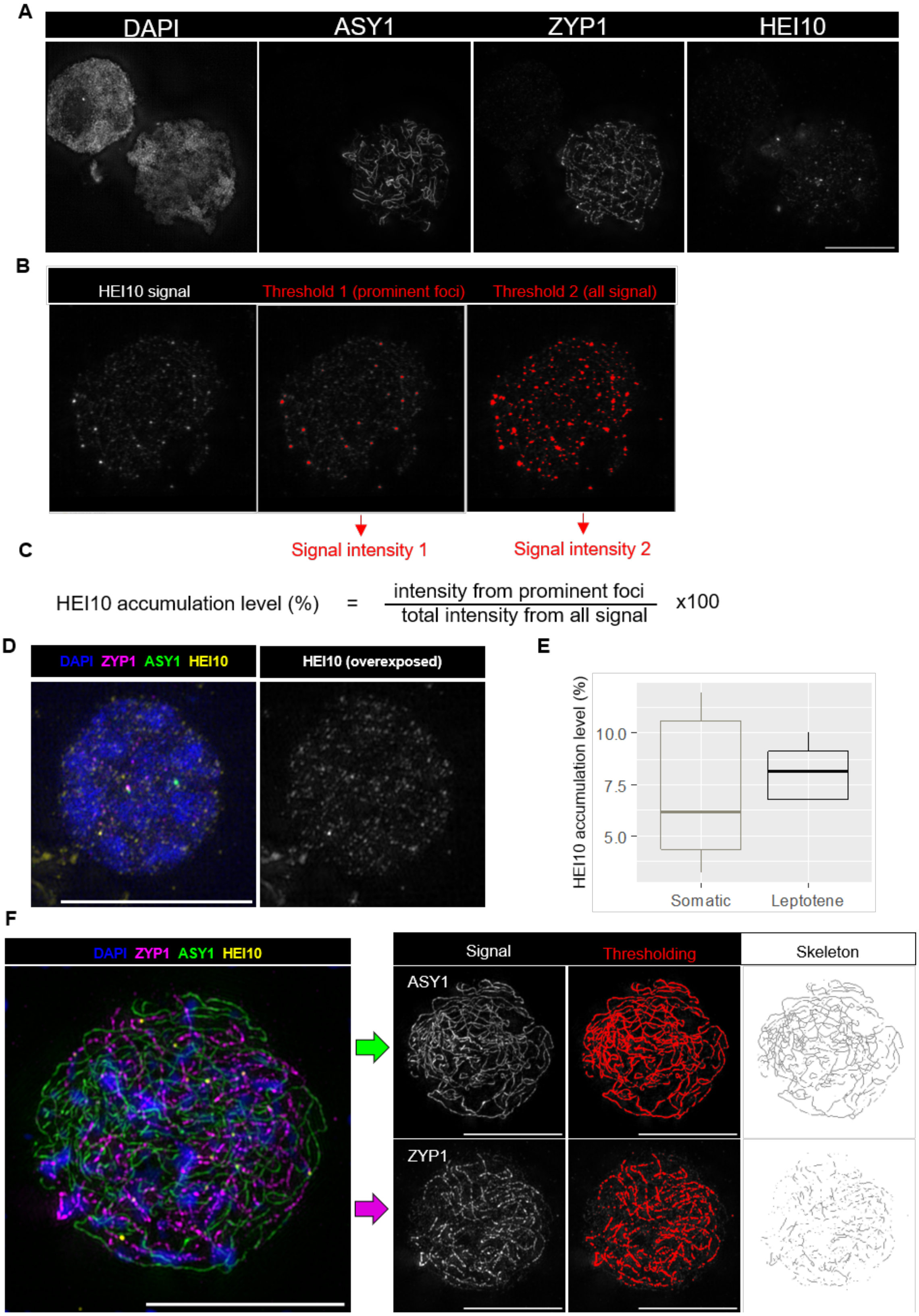
A
DAPI
ASY1
ZYP1
HEI10
B
HEI10 signal
Threshold 1 (prominent foci)
Threshold 2 (all signal)
Signal intensity 1
Signal intensity 2
C
HEI10 accumulation level (%) = intensity from prominent foci / total intensity from all signal x100
D
DAPI ZYP1 ASY1 HEI10
HEI10 (overexposed)
E
HEI10 accumulation level (%)
10.0
7.5
5.0
Somatic
Leptotene
F
DAPI ZYP1 ASY1 HEI10
Signal
Thresholding
Skeleton
ASY1
ZYP1

Fig. S2. Quantification of HEI10 accumulation level and extent of asynapsis. (A) Fluorescence staining for two cells: a somatic (only DAPI stains it substantially) with a meiotic cell (with specific staining for DAPI, ASY1, ZYP1 and HEI10) at the lower right. (B) Example images showing the HEI10 channel of one imaged cell displaying the two thresholds assigned in Fiji: Threshold 1 (Yen method (72)) detects the relatively prominent foci, whereas Threshold 2 (Moments method (73)) detects the total HEI10 signal. (C) How HEI10 accumulation level is calculated from the values of signal intensity from Threshold 1 and Threshold 2. (D) The background signal from HEI10 staining in a somatic cell (signal has been overexposed for display). (E) The HEI10 accumulation values of leptotene and somatic cells (n=11, calculated in NEO-4X cells) where no HEI10 loading is expected. (F) Illustrates how we quantify ASY1 and ZYP1 length a NEO-4X cells with extensive asynapsis in Fiji. Each channel is processed separately. After thresholding, the detected signal is skeletonized and measured. This example illustrates how ASY1 signal generates high quality skeletons whereas ZYP1 signal, which often displays a dotted pattern, yields discontinuous skeletons, severely underestimating its real length in automated measurements.

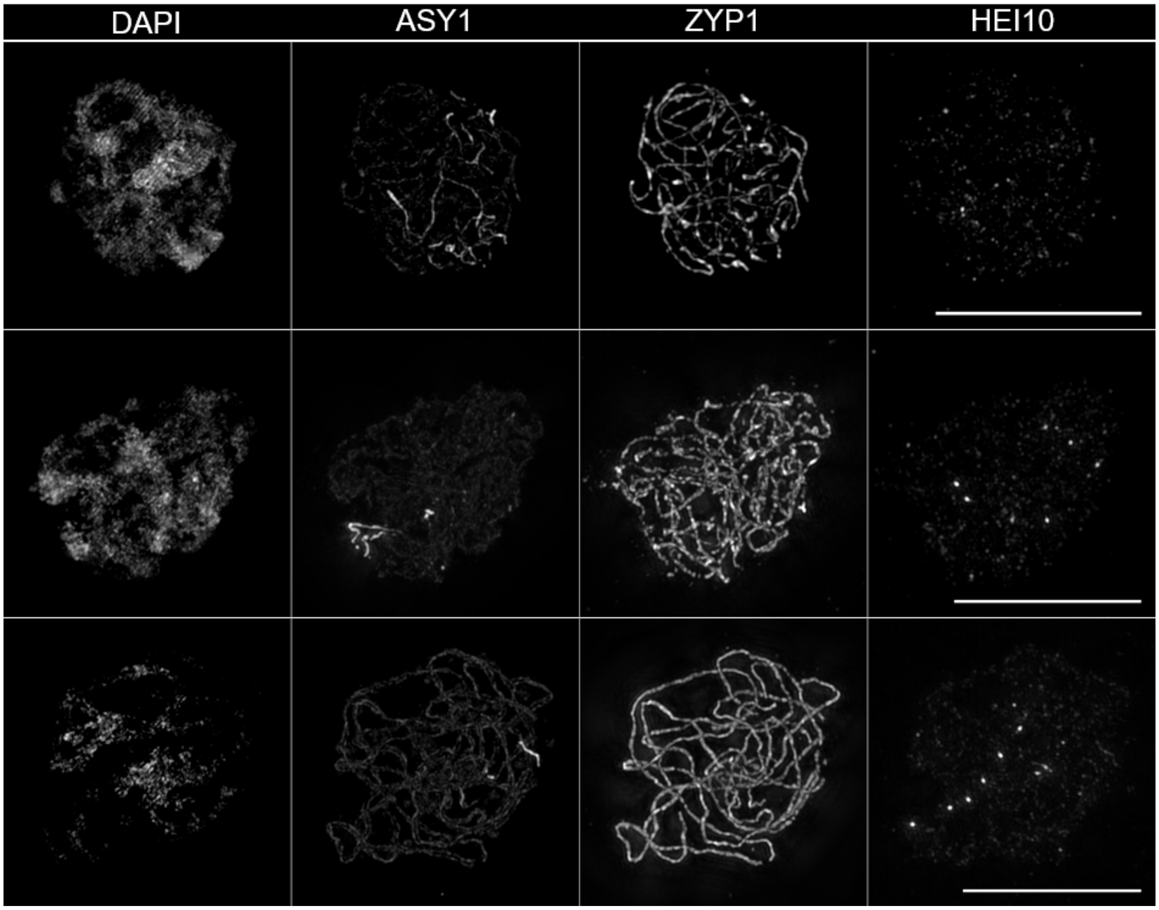

Fig. S3. Separate channels for the examples of DIP-2X cells shown in Fig. 3. The HEI10 accumulation levels are 6.7, 36.0 and 47.5%, from top to bottom.

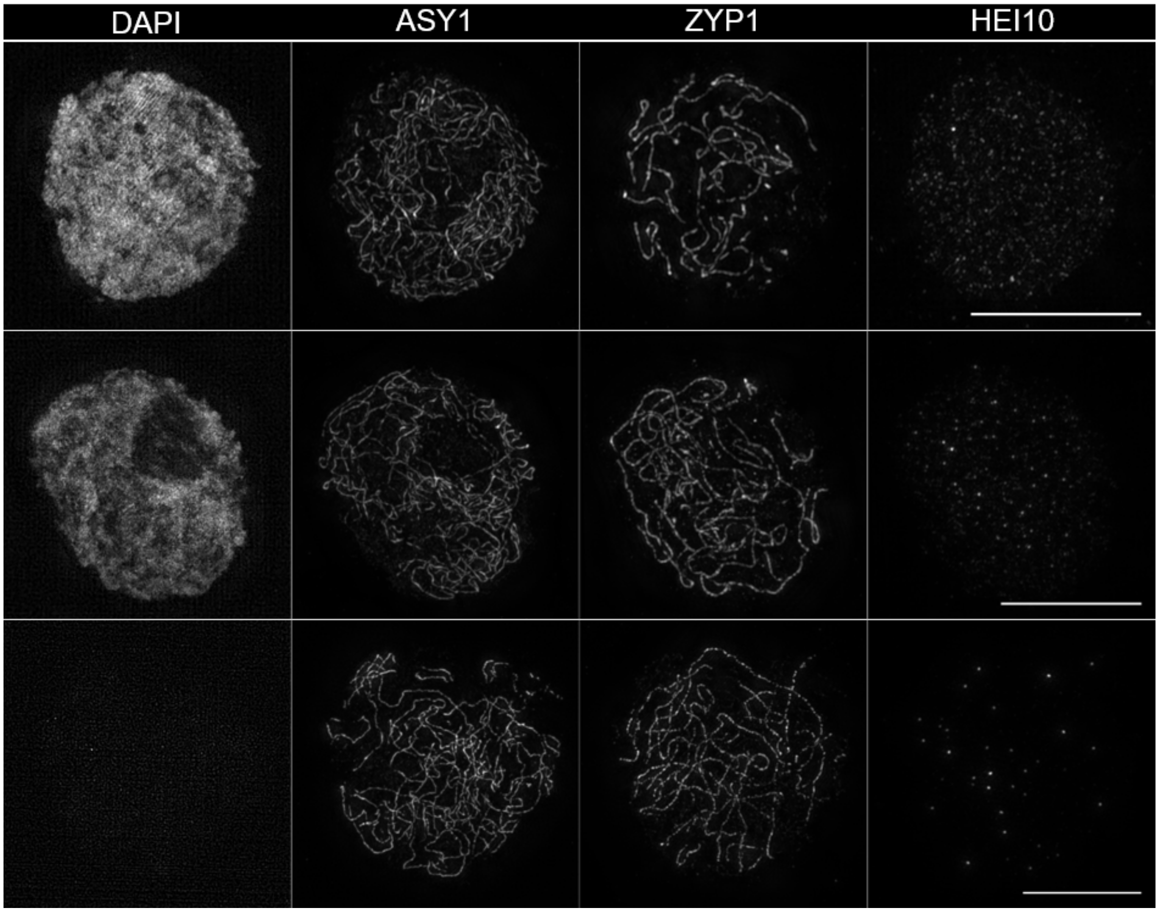

Fig. S4. Separate channels for the examples of NEO-4X cells shown in Fig. 3. The HEI10 accumulation levels are 3.9, 33.1 and 100%, from top to bottom.

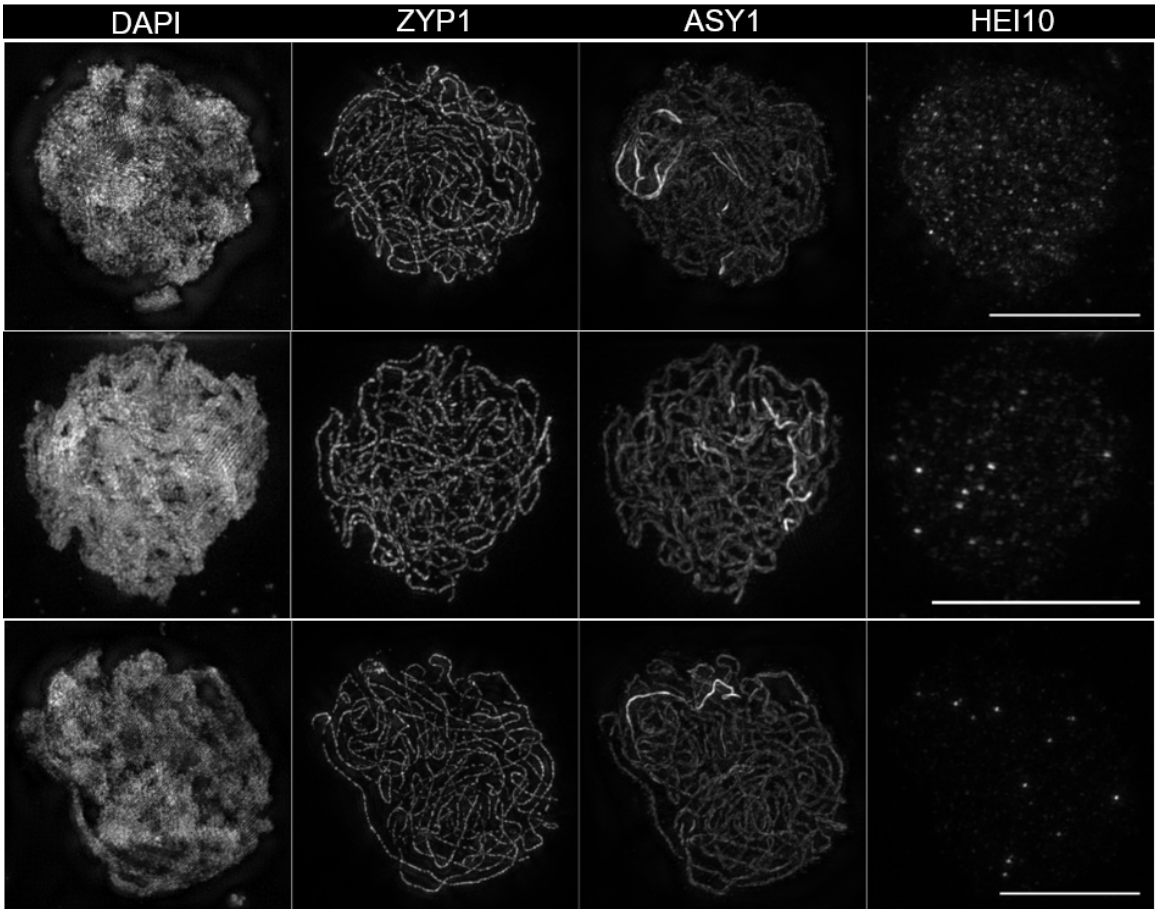

Fig. S5. Separate channels for the examples of HYB-4X cells shown in Fig. 3. The HEI10 accumulation levels are 9.8, 38.7 and 71.7% from top to bottom.

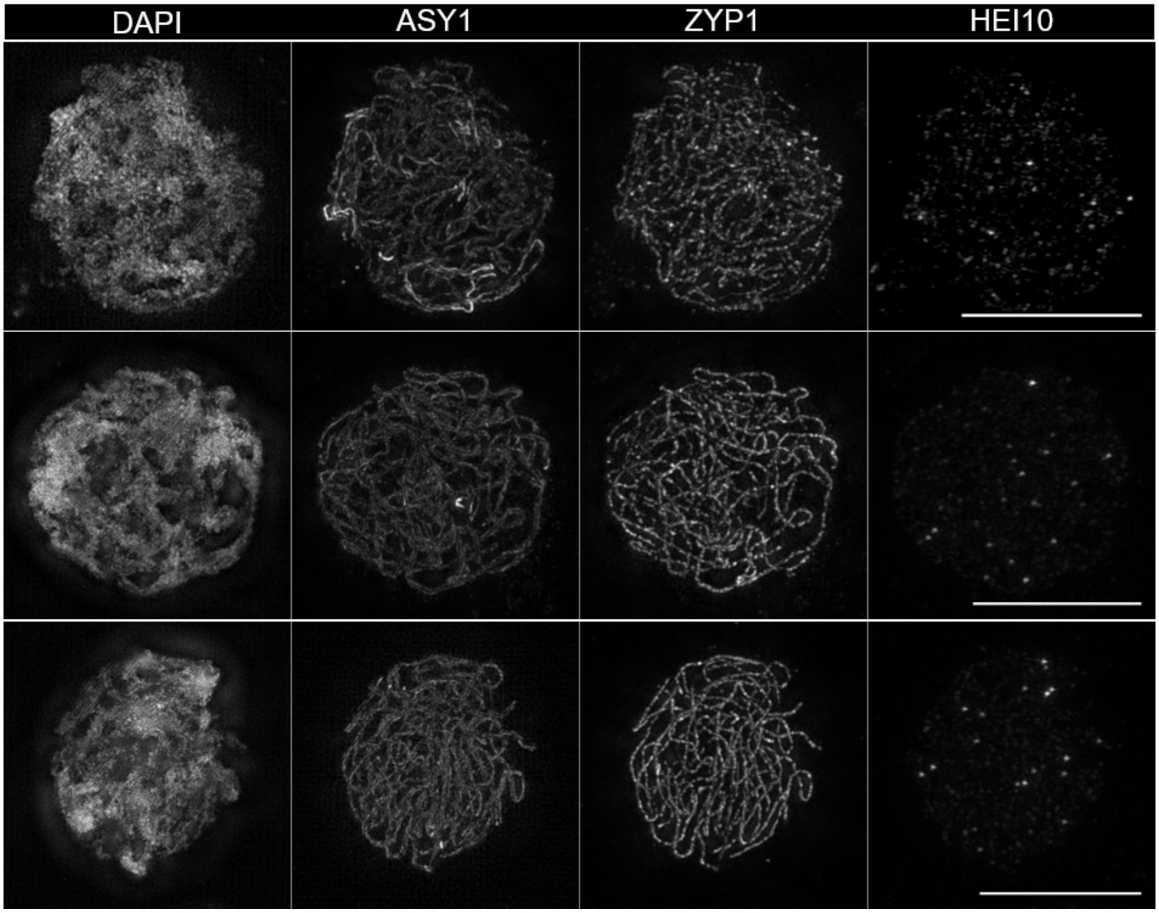

Fig. S6. Separate channels for the examples of NEO-4X cells shown in Fig. 3. The HEI10 accumulation levels are 2.3, 34.2 and 60.7% from top to bottom.

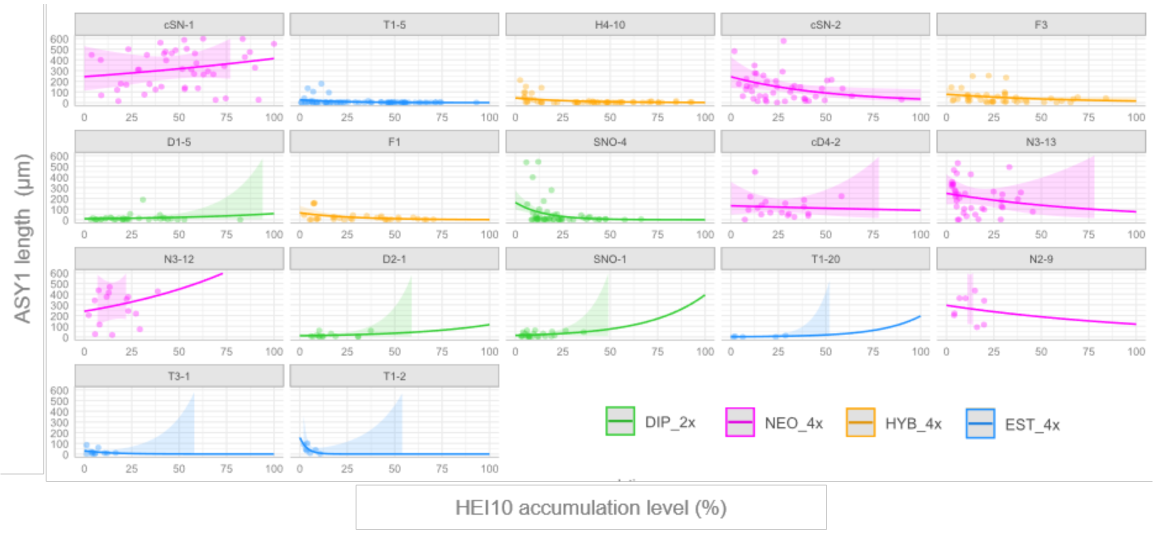

Fig. S7. Synaptic behavior of multiple individuals.

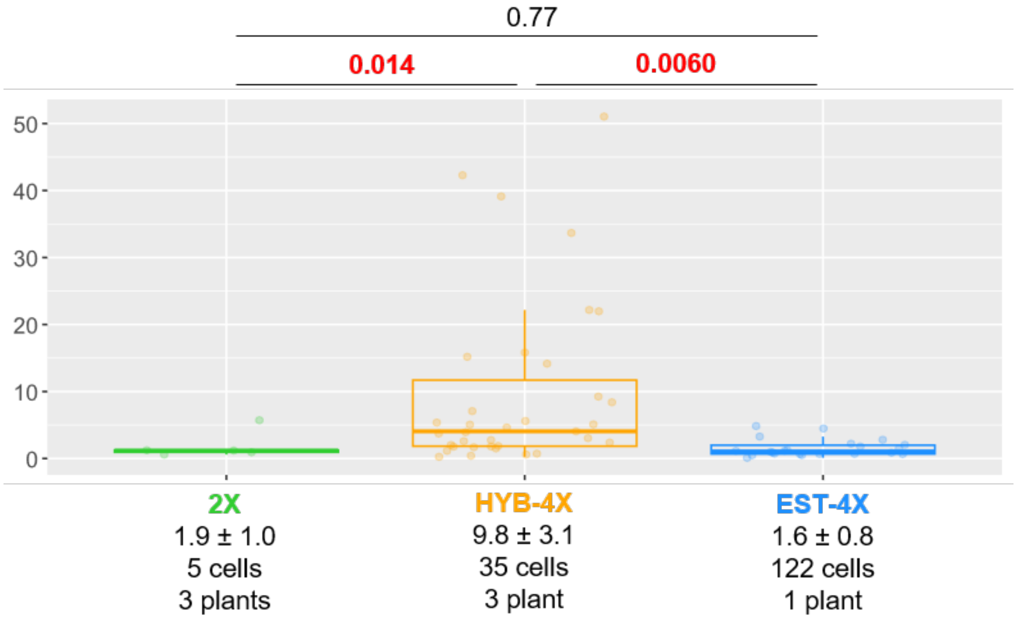

Fig. S8. Final levels of asynapsis. This is an alternative to the graph shown in figure 3D, but without NEO-4X in order to have a scale that allows better visualization of the data of the genotypes with very low levels of asynapsis. P-values are indicated on top of the plots for each comparison (according to Wald's test on Gamma-GLMM coefficients). Significant p-values were highlighted in red. Mean values (Mean ± SE) and sample sizes are indicated in the lower part of the plots.

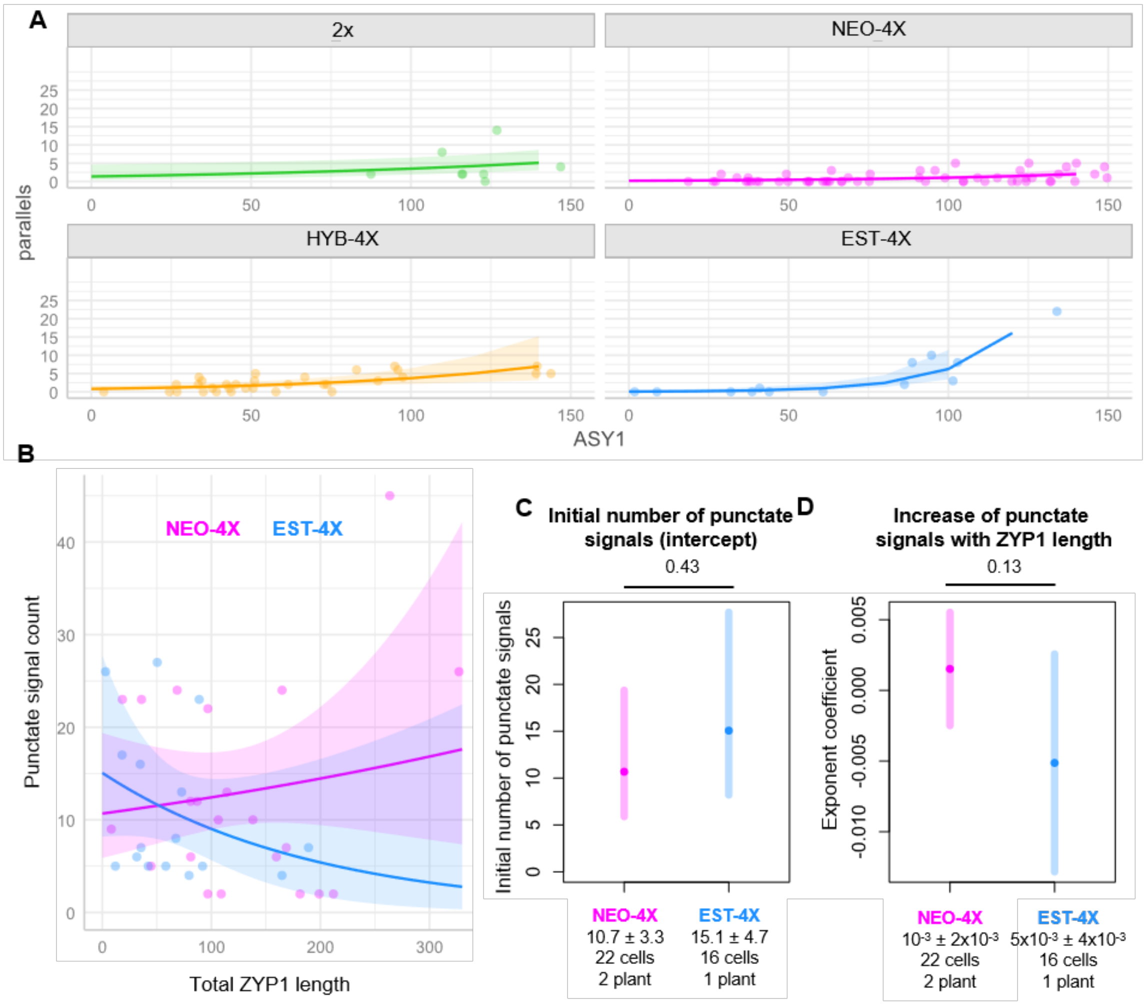

Fig. S9. Statistical models for synapsis defects. (A) Plots of the data and fitted trendlines predicted by a negative-binomial-GLMM explaining the change in the number of instances of parallel axes as the length of ASY1 signal increases for the four genotypes. (B) Plots of the data and the trendlines predicted by a negative binomial-GLMM for the change in the number of punctate ZYP1 signals. (C) and (D) are plots showing differences predicted by the fitted model in the intercept for both genotypes (i.e. in the initial number of punctate signals, when ZYP1 length is zero, in (C) the exponent coefficient (i.e. the change of the number of punctate signals as ZYP1 length increases). P-values are indicated on top of the plots for each comparison (according to Wald's test on Poisson-GLMM coefficients). Significant p-values were highlighted in red. Mean values (Mean ± SE) and sample sizes are indicated in the lower part of the plots.

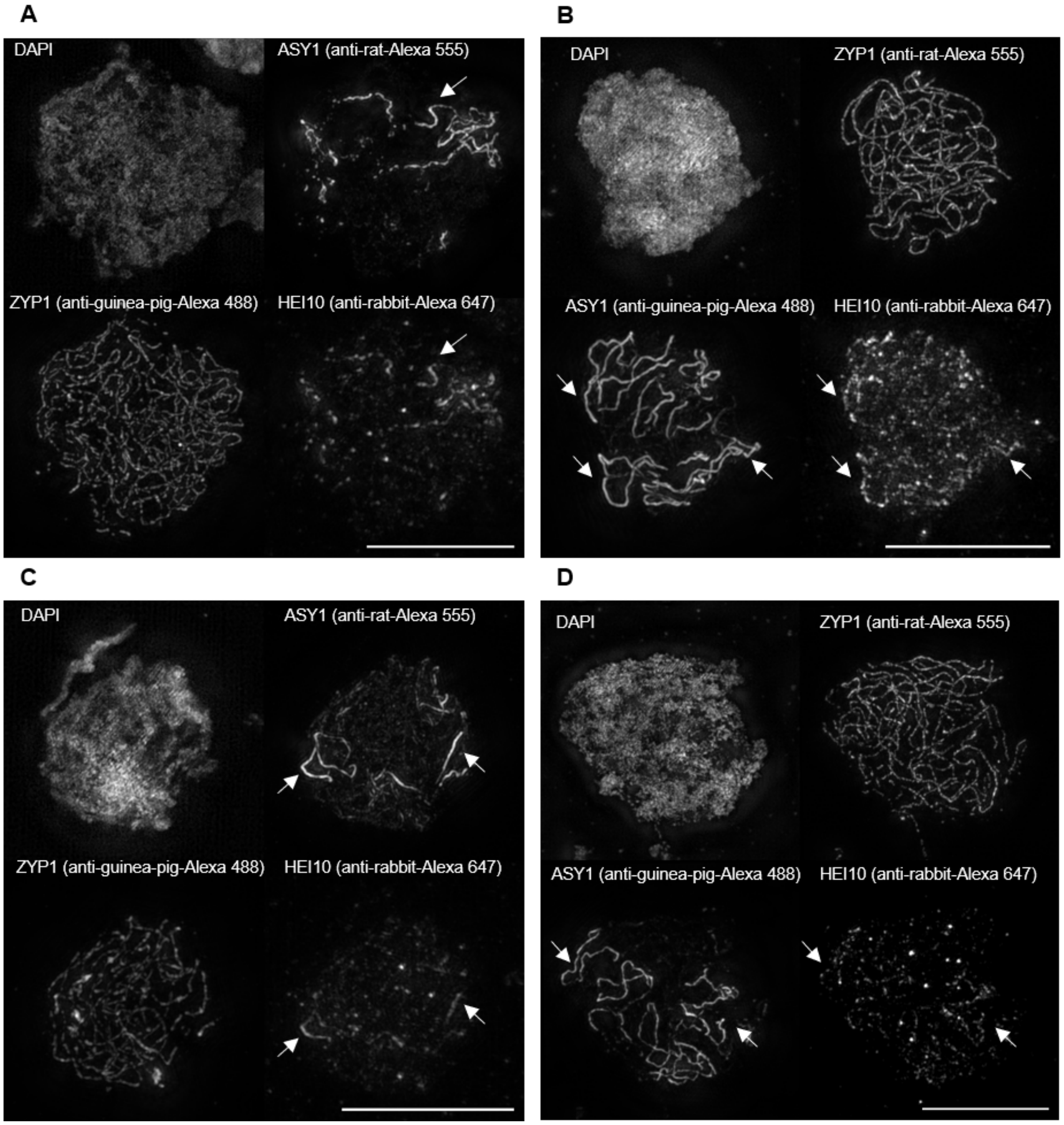

Fig. S10. HEI10-ASY1 overlapping revealed by different combination of antibodies. (A) and (C) show immunostaining with one combination of primary and secondary antibodies whereas (B) and (D) shows a different combination.

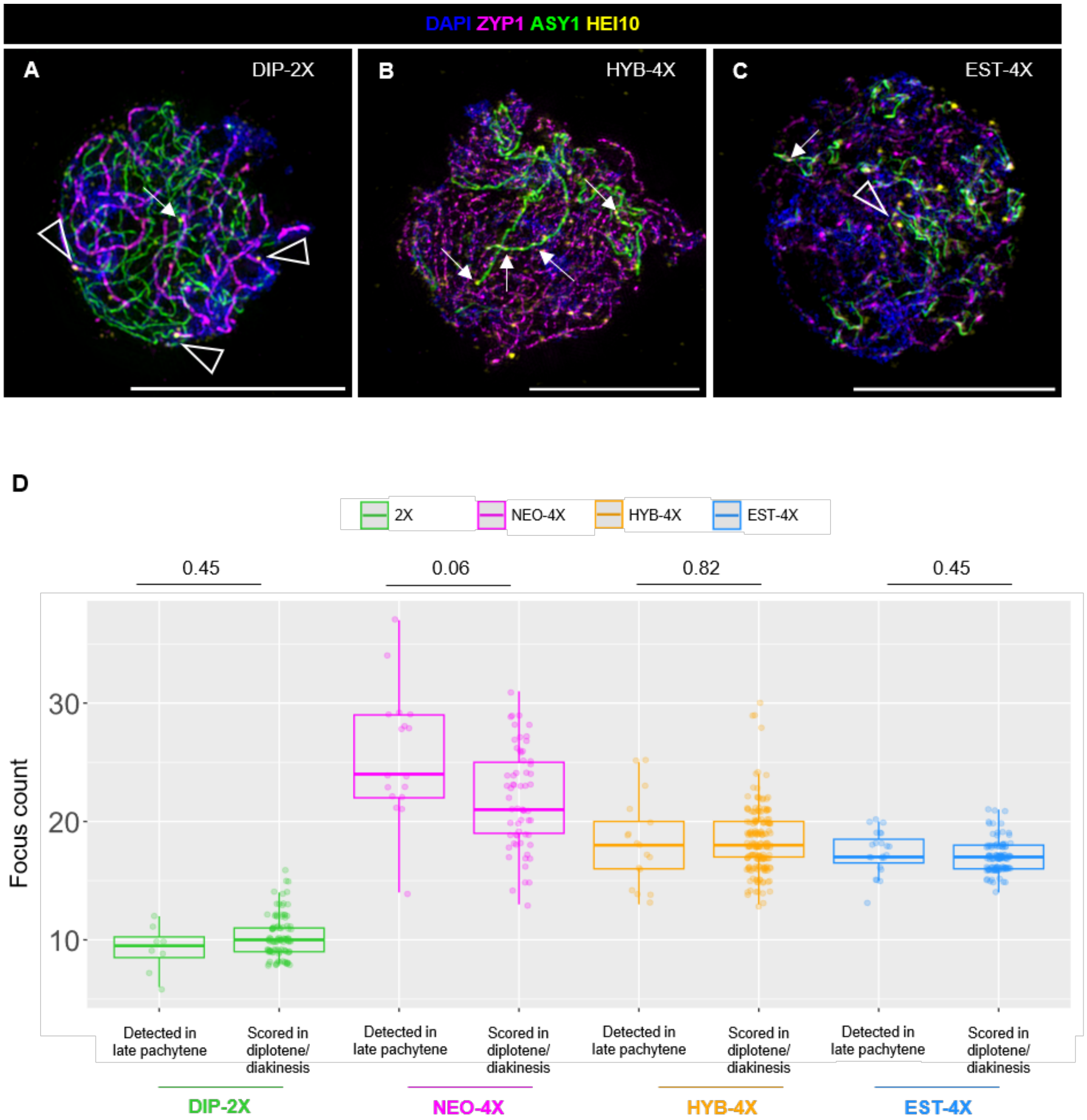

Fig. S11. Effect of asynapsis on HEI10 localization and crossover number. (A), (B) and (C) are examples of 2X, HYB-4X and EST-4X cells (respectively) showing limited preference of prominent HEI10 foci for synapsed regions. Hollow arrowheads point at some examples of prominent HEI10 foci localized to synapsed regions, whereas filled arrows point at some examples of foci localized to unsynapsed regions (D) Comparisons of crossover number estimations based on HEI10 focus counts detected in late pachytene SIM images and manually scored in diplotene/pachytene cells for each genotype. P-values are indicated on top of the plots for each comparison (according to t-tests, except for the EST-4X comparison, which was done using the Mann-Whitney U test).

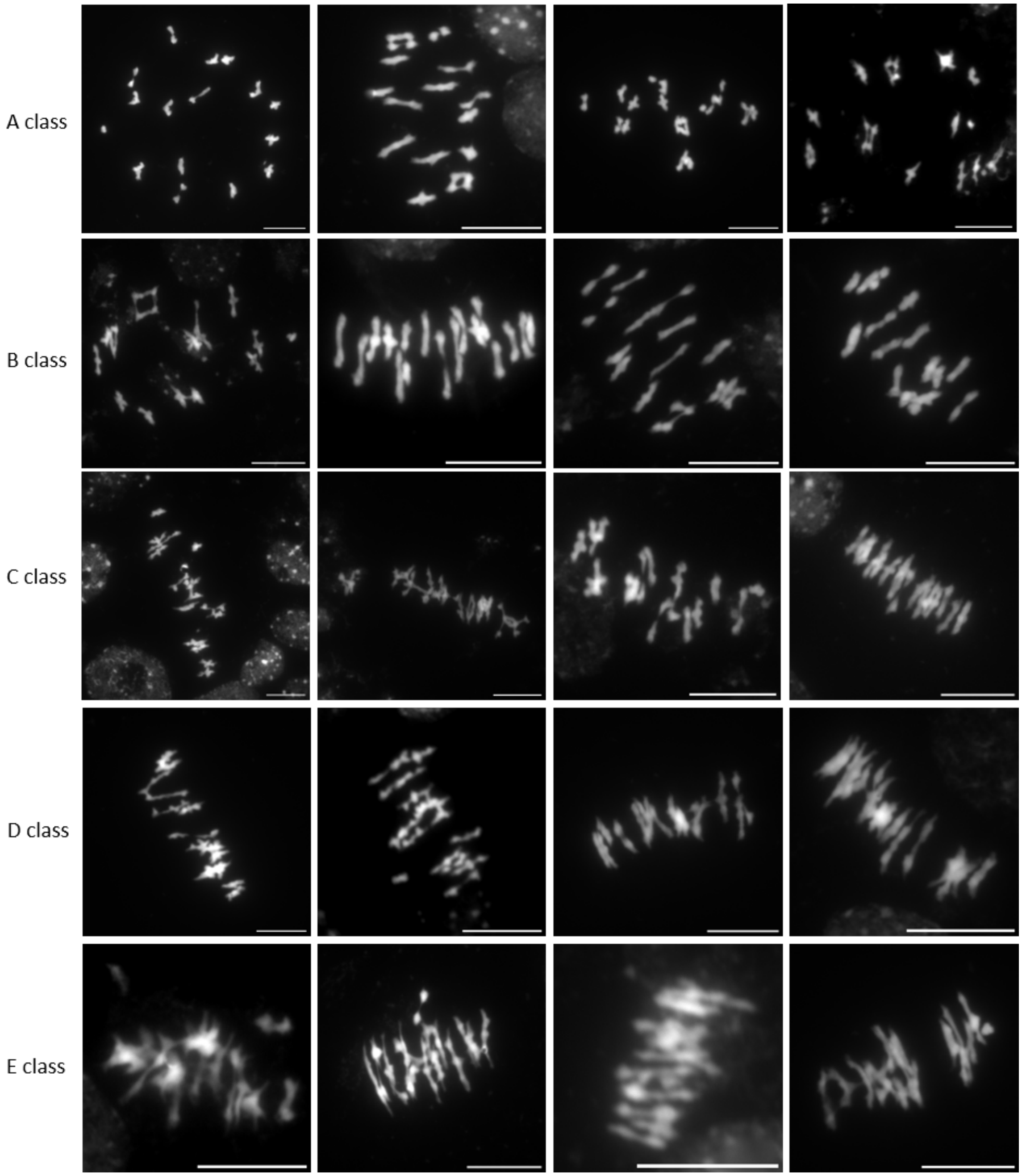

Fig. S12. Scorability classes in metaphase I. Examples of metaphase I cells assigned to different "scorability" classes named A, B, C, D, or E, depending on the quality of the spread and the confidence in the count, with A being the gold standard and E the least reliable.

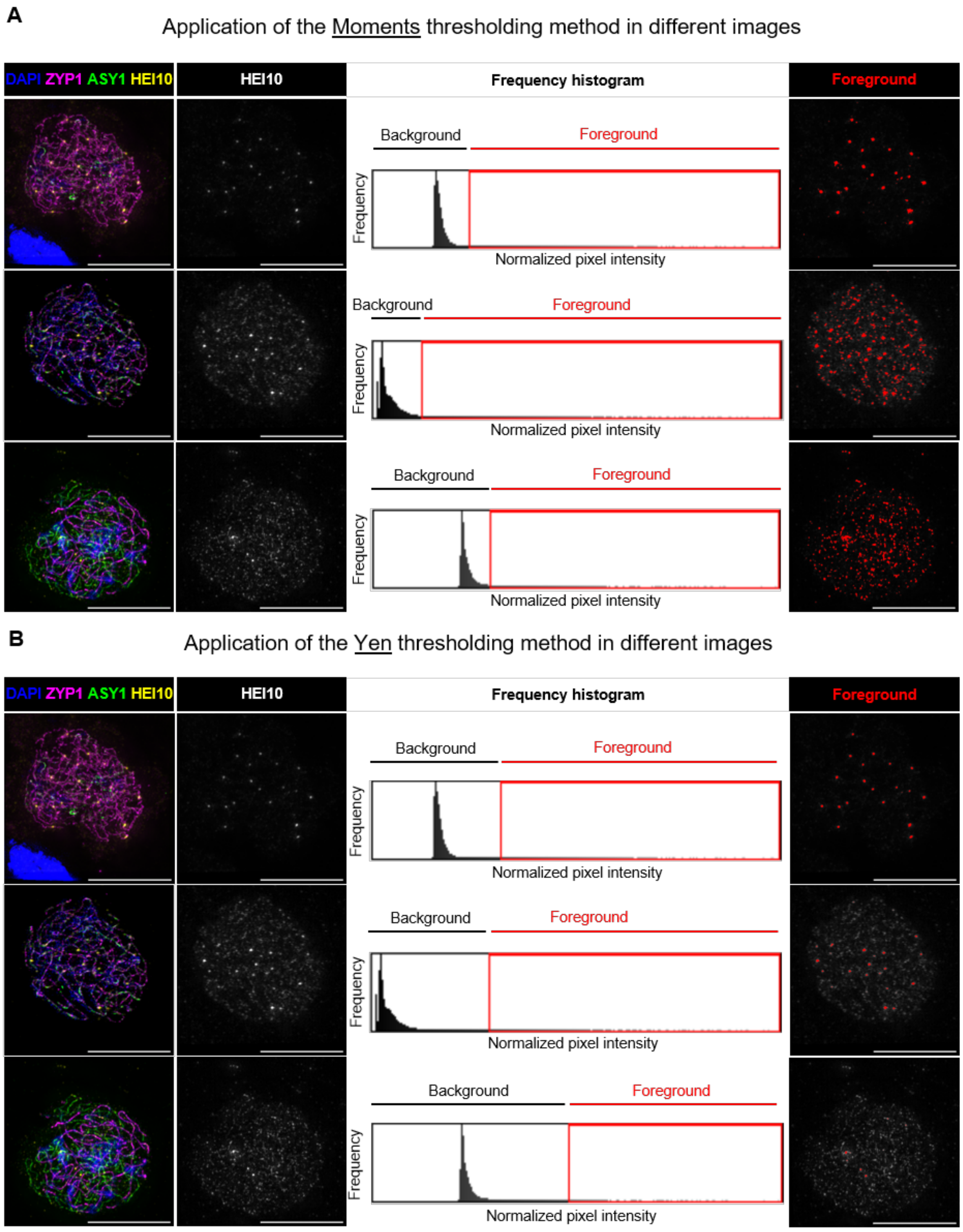
A
Application of the Moments thresholding method in different images
DAPI ZYP1 ASY1 HEI10
HEI10
Frequency histogram
Foreground
Background
Foreground
Frequency
Normalized pixel intensity
B
Application of the Yen thresholding method in different images
DAPI ZYP1 ASY1 HEI10
HEI10
Frequency histogram
Foreground
Background
Foreground
Frequency
Normalized pixel intensity

Fig. S13. Fiji/ImageJ thresholds. This figure illustrates how thresholding methods available in Fiji/ImageJ operate in the HEI10 channel of the same three example images. For each image, Fiji generates a frequency histogram based on the distribution of the pixel intensities. Then, based on this distribution different algorithms are applied depending on the thresholding methods. The Yen method (A) finds the criterion to separate foreground (in red) from background (in black) by maximizing the entropy of the image, whereas the Moments method (B) is optimized to preserve the statistical properties (or moments, namely, mean, variance, skewness, and kurtosis) of the original image in the thresholded result. Importantly these examples illustrate how thresholds are based on relative intensity and, when applied to different images, the same thresholding method will find different intensity cutoffs to separate foreground from background.

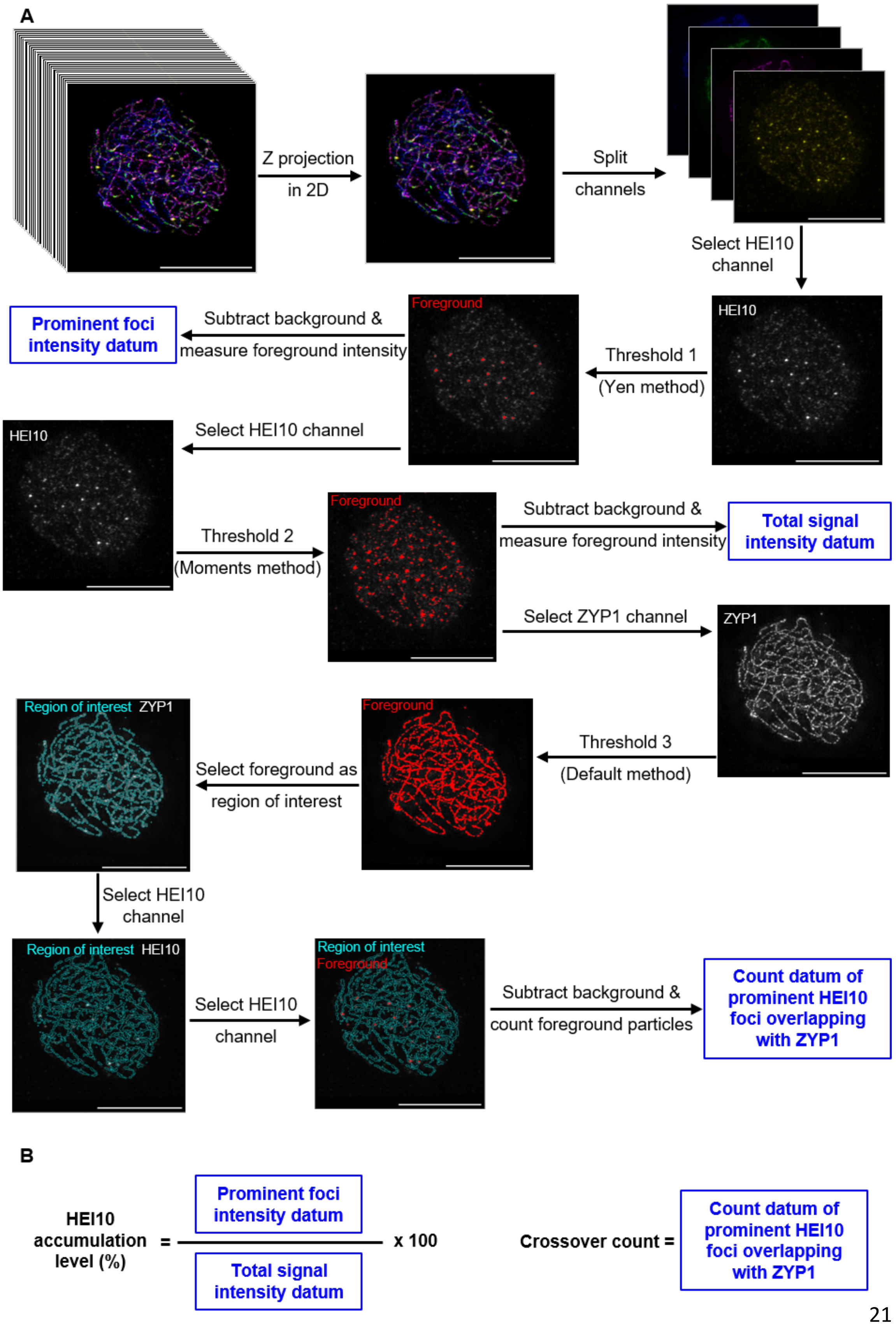
A
Z projection
in 2D
Split
channels
Select HEI10
channel
HEI10
Threshold 1
(Yen method)
Foreground
Subtract background &
measure foreground intensity
Prominent foci
intensity datum
Select HEI10 channel
HEI10
Threshold 2
(Moments method)
Foreground
Subtract background &
measure foreground intensity
Total signal
intensity datum
Select ZYP1 channel
ZYP1
Threshold 3
(Default method)
Foreground
Select foreground as
region of interest
Region of interest
ZYP1
Select HEI10
channel
Region of interest
HEI10
Select HEI10
channel
Region of interest
Foreground
Subtract background &
count foreground particles
Count datum of
prominent HEI10
foci overlapping
with ZYP1
B
HEI10
accumulation
level (%)
=
Prominent foci
intensity datum
Total signal
intensity datum
x 100
Crossover count =
Count datum of
prominent HEI10
foci overlapping
with ZYP1

.Fig. S14. Workflow of Macro 1. (A) diagram of how Macro 1 operates on SIM images to obtain three different analysis outputs (highlighted in blue): prominent foci intensity, total signal intensity, and the count of prominent HEI10 foci overlapping with ZYP1. (B) Shows how the data outputs for each image are used in the R Script to calculate the HEI10 accumulation level and the crossover count (only in cells where the HEI10 accumulation level surpasses our calculated cutoff, as explained in the results).

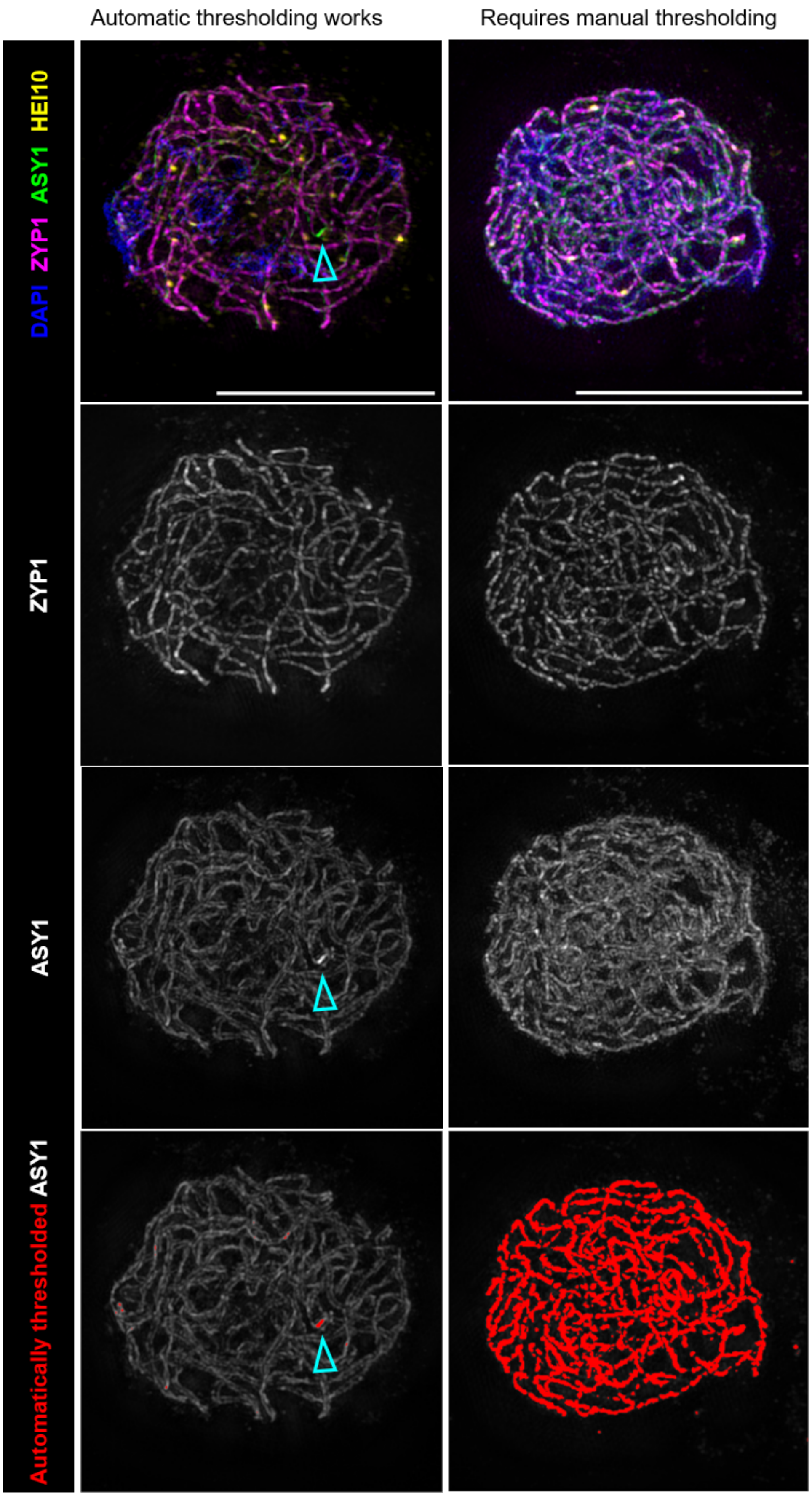
Automatic thresholding works
Requires manual thresholding
DAPI ZYP1 ASY1 HEI10
ZYP1
ASY1
Automatically thresholded ASY1

Fig. S15. Automatic thresholding for ASY1 signal. Examples of two whose ASY1 signals are detected differently by Yen threshold. The first cell (left) displays complete synapsis and the threshold does not detect the dim ASY1 signal characteristic of a remodeled axis after synapsis. A miniscule artifact or synaptic imperfection (cyan arrowhead), makes the threshold to detect it yielding negligible ASY1 length measurement. By contrast the other cell (right), shows also full synapsis but there is no artifact or synaptic imperfection. Therefore since the threshold operates with relative intensities (72), detects all the dim ASY1 signal. This causes a massive overestimation of the ASY1 length where massive asynapsis measurements are given for fully synapsed cell. Fortunately, these cells are rare and easily detected with Macro 3 as described in Supporting Information Text.

Table S1. Plant material used for this study. It is indicated whether the plant was colchicine-treated and how each plant was analyzed; either with epifluorescence microscopy (metaphase I spreads plus late prophase I HEI10 immunostaining) or SIM (for prophase I dynamics analysis).

Plant name	Genotype	Colchicine-treated?	Use
D1-5	2X	No	Epifluorescence
D1-5	2X	No	SIM
D2-1	2X	No	SIM
SNO-1	2X	No	SIM
SNO-4	2X	No	SIM
cD4-2	NEO-4X	Yes	SIM
cSN-1	NEO-4X	Yes	SIM
cSN-10-1	NEO-4X	Yes	Epifluorescence
cSN-2	NEO-4X	Yes	SIM
N1-5	NEO-4X	No	Epifluorescence
N2-1	NEO-4X	No	Epifluorescence
N2-2	NEO-4X	No	Epifluorescence
N2-5	NEO-4X	No	Epifluorescence
N2-6	NEO-4X	No	Epifluorescence
N2-9	NEO-4X	No	Epifluorescence
N2-9	NEO-4X	No	SIM
N3-4	NEO-4X	No	Epifluorescence
N3-12	NEO-4X	No	SIM
N3-13	NEO-4X	No	Epifluorescence + SIM
F1	HYB-4X	No	SIM
F1-2-3	HYB-4X	No	Epifluorescence
F2-3-3	HYB-4X	No	Epifluorescence
F3	HYB-4X	No	SIM
F3-1	HYB-4X	No	Epifluorescence
F3-4-1	HYB-4X	No	Epifluorescence
H4-10	HYB-4X	No	Epifluorescence
H4-10	HYB-4X	No	SIM
H4-9	HYB-4X	No	Epifluorescence
T1-2	EST-4X	No	SIM
T1-20	EST-4X	No	Epifluorescence + SIM
T1-23	EST-4X	No	Epifluorescence
T1-5	EST-4X	No	Epifluorescence + SIM
T2-1	EST-4X	No	Epifluorescence
T2-10	EST-4X	No	Epifluorescence
T2-3	EST-4X	No	Epifluorescence
T3-1	EST-4X	No	SIM

SI References

1. G. S. Roeder, Meiotic chromosomes: it takes two to tango. *Genes Dev.* **11**, 2600–21 (1997).
2. L. Grandont, E. Jenczewski, A. Lloyd, Meiosis and Its Deviations in Polyploid Plants. *Cytogenet. Genome Res.* **14**, 171–184 (2013).
3. A. Gonzalo, All Ways Lead to Rome—Meiotic Stabilization Can Take Many Routes in Nascent Polyploid Plants. *Genes (Basel).* **13** (2022).
4. J. Szostak, T. Orr-Weaver, R. Rothstein, F. Stahl, The double-strand-break repair model for recombination. *Cell* **33**, 25–35 (1983).
5. C. Morgan, A. Nayak, N. Hosoya, G. R. Smith, C. Lambing, "Meiotic chromosome organization and its role in recombination and cancer" in *Current Topics in Developmental Biology*, 1st Ed., (Elsevier Inc., 2023), pp. 91–126.
6. D. Zickler, N. Kleckner, Meiotic chromosomes: integrating structure and function. *Annu. Rev. Genet.* **33**, 603–754 (1999).
7. A. Caryl, S. Armstrong, G. Jones, F. Franklin, A homologue of the yeast HOP1 gene is inactivated in the Arabidopsis meiotic mutant asy1. *Chromosoma* **109**, 62–71 (2000).
8. C. Lambing, P. C. Kuo, A. J. Tock, S. D. Topp, I. R. Henderson, ASY1 acts as a dosage-dependent antagonist of telomere-led recombination and mediates crossover interference in Arabidopsis. *Proc. Natl. Acad. Sci. U. S. A.* **117**, 13647–13658 (2020).
9. J. C. Fung, B. Rockmill, M. Odell, G. S. Roeder, Imposition of crossover interference through the nonrandom distribution of synapsis initiation complexes. *Cell* **116**, 795–802 (2004).
10. L. Chelysheva, *et al.*, The Arabidopsis HEI10 is a new ZMM protein related to Zip3. *PLoS Genet.* **8** (2012).
11. A. Reynolds, *et al.*, RNF212 is a dosage-sensitive regulator of crossing-over during mammalian meiosis. *Nat. Genet.* **45**, 269–78 (2013).
12. P. A. Ziolkowski, *et al.*, Natural variation and dosage of the HEI10 meiotic E3 ligase control Arabidopsis crossover recombination. *Genes Dev.* **31**, 306–317 (2017).
13. A. Gonzalo, *et al.*, Reducing MSH4 copy number prevents meiotic crossovers between non-homologous chromosomes in Brassica napus. *Nat. Commun.* **10**, 2354 (2019).
14. C. Morgan, *et al.*, Diffusion-mediated HEI10 coarsening can explain meiotic crossover positioning in Arabidopsis. *Nat. Commun.* **12**, 4674 (2021).
15. L. Zhang, W. Stauffer, D. Zwicker, A. F. Dernburg, @ Affiliations, Crossover patterning through kinase-regulated condensation and coarsening of recombination nodules. *bioRxiv*, 2021.08.26.457865 (2021).
16. C. M. Anderson, A. Oke, P. Yam, T. Zhuge, J. C. Fung, Reduced Crossover Interference and Increased ZMM-Independent Recombination in the Absence of Tel1/ATM. *PLoS Genet.* **11**, 1–27 (2015).
17. L. Zhang, E. Espagne, A. De Muyt, D. Zickler, N. E. Kleckner, Interference-mediated synaptonemal complex formation with embedded crossover designation. *Proc. Natl. Acad. Sci. U. S. A.* **111**, E5059–E5068 (2014).
18. D. E. Libuda, S. Uzawa, B. J. Meyer, A. M. Villeneuve, Meiotic chromosome structures constrain and respond to designation of crossover sites. *Nature* **502**, 703–706 (2013).
19. Y. Duroc, *et al.*, The Kinesin AtPSS1 Promotes Synapsis and is Required for Proper Crossover Distribution in Meiosis. *PLoS Genet* **10** (2014).
20. C. K. Cahoon, J. M. Helm, D. E. Libuda, Synaptonemal Complex Central Region Proteins Promote Localization of Pro-crossover Factors to Recombination Events During Caenorhabditis elegans Meiosis. *Genetics* **213**, 395–409 (2019).
21. J. A. Fozard, C. Morgan, M. Howard, Coarsening dynamics can explain meiotic crossover patterning in both the presence and absence of the synaptonemal complex. *Elife* **12**, 1–25 (2023).
22. K. Wang, *et al.*, The role of rice HEI10 in the formation of meiotic crossovers. *PLoS Genet.*

8 (2012).
23. L. Grandont, *et al.*, Homoeologous Chromosome Sorting and Progression of Meiotic Recombination in Brassica napus: Ploidy Does Matter! *Plant Cell* **26**, 1448–1463 (2014).
24. S. D. Desjardins, *et al.*, MutS homologue 4 and MutS homologue 5 Maintain the Obligate Crossover in Wheat Despite Stepwise Gene Loss following Polyploidization. *Plant Physiol.* **183**, 1545–1558 (2020).
25. B. Arnold, S.-T. Kim, K. Bomblies, Single Geographic Origin of a Widespread Autotetraploid Arabidopsis arenosa Lineage Followed by Interploidy Admixture. *Mol. Biol. Evol.* **32**, 1382–95 (2015).
26. C. Morgan, *et al.*, Evolution of crossover interference enables stable autopolyploidy by ensuring pairwise partner connections in Arabidopsis arenosa. *Curr. Biol.*, 1–14 (2021).
27. M. A. Prusicki, *et al.*, Live cell imaging of meiosis in arabidopsis thaliana. *Elife* **8**, 1–31 (2019).
28. L. Yant, *et al.*, Meiotic Adaptation to Genome Duplication in Arabidopsis arenosa. *Curr. Biol.*, 1–6 (2013).
29. A. Gonzalo, P. Parra-Nunez, A. L. Bachmann, E. Sanchez-Moran, K. Bomblies, Partial cytological diploidization of neoautotetraploid meiosis by induced cross-over rate reduction. *Proc. Natl. Acad. Sci.* **120**, 2017 (2023).
30. K. Bomblies, G. Jones, C. Franklin, D. Zickler, N. Kleckner, The challenge of evolving stable polyploidy: could an increase in “crossover interference distance” play a central role? *Chromosoma* **125**, 287–300 (2016).
31. D. Kostoff, Fertility and chromosome length. *J. Hered.* **31**, 33–34 (1940).
32. L. Chelysheva, L. Grandont, M. Grelon, Immunolocalization of meiotic proteins in Brassicaceae: method 1. *Methods Mol Biol*, 990:93 (2013).
33. C. Morgan, H. Zhang, C. E. Henry, C. F. H. Franklin, K. Bomblies, Derived alleles of two axis proteins affect meiotic traits in autotetraploid Arabidopsis arenosa. *Proc. Natl. Acad. Sci. U. S. A.* **117**, 8980–8988 (2020).
34. M. Ito, *et al.*, Distinct and interdependent functions of three RING proteins regulate recombination during mammalian meiosis. *bioRxiv*, 2023.11.07.566091 (2023).
35. J. Varas, *et al.*, Absence of SUN1 and SUN2 proteins in Arabidopsis thaliana leads to a delay in meiotic progression and defects in synapsis and recombination. *Plant J.* **81**, 329–46 (2015).
36. M. Castellani, *et al.*, Meiotic recombination dynamics in plants with repeat-based holocentromeres shed light on the primary drivers of crossover patterning. *Nat. plants* **10**, 423–438 (2024).
37. S. Agarwal, G. S. Roeder, Zip3 Provides a Link between Recombination Enzymes and Synaptonemal Complex Proteins. **102**, 245–255 (2000).
38. N. Bhalla, D. J. Wynne, V. Jantsch, A. F. Dernburg, ZHP-3 acts at crossovers to couple meiotic recombination with synaptonemal complex disassembly and bivalent formation in C. elegans. *PLoS Genet.* **4** (2008).
39. M. T. Jahns, *et al.*, Crossover Localisation Is Regulated by the Neddylation Posttranslational Regulatory Pathway. *PLoS Biol* **12** (2014).
40. S. Mlynarczyk-Evans, B. Roelens, A. M. Villeneuve, Evidence That Masking of Synapsis Imperfections Counterbalances Quality Control to Promote Efficient Meiosis. *PLoS Genet.* **9**, 14–16 (2013).
41. C. Nibau, A. Evans, H. King, D. W. Phillips, A. Lloyd, Homoeologous crossovers are distally biased and underlie genomic instability in first-generation neo-allopolyploid Arabidopsis suecica. *New Phytol.* (2024) https:/doi.org/10.1111/nph.20095.
42. C. Nibau, *et al.*, Meiosis in allopolyploid Arabidopsis suecica. *Plant J.* **111**, 1110–1122 (2022).
43. L. von Diezmann, C. Bristow, O. Rog, Diffusion within the synaptonemal complex can account for signal transduction along meiotic chromosomes. *bioRxiv* (2024).
44. M. Martinez-Garcia, C. I. White, F. C. H. Franklin, E. Sanchez-Moran, The role of topoisomerase II in DNA repair and recombination in arabidopsis thaliana. *Int. J. Mol. Sci.*

22 (2021).
45. K. Voelkel-meiman, C. Johnston, Y. Thappeta, Separable Crossover-Promoting and Crossover-Constraining Aspects of Zip1 Activity during Budding Yeast Meiosis. **5**, 1–39 (2015).
46. X. Mu, H. Murakami, N. Mohibullah, S. Keeney, Chromosome-autonomous feedback down-regulates meiotic DNA break competence upon synaptonemal complex formation. *Genes Dev.* **34**, 1605–1618 (2020).
47. S. G. Gordon, L. E. Kursel, K. Xu, O. Rog, Synaptonemal Complex dimerization regulates chromosome alignment and crossover patterning in meiosis. *PLoS Genet.* **17**, e1009205 (2021).
48. L. I. Láscarez-Lagunas, *et al.*, ATM/ATR kinases link the synaptonemal complex and DNA double-strand break repair pathway choice. *Curr. Biol.* **32**, 4719-4726.e4 (2022).
49. L. Capilla-Pérez, *et al.*, The synaptonemal complex imposes crossover interference and heterochiasmy in Arabidopsis. *Proc. Natl. Acad. Sci. U. S. A.* **118**, 1–11 (2021).
50. M. G. France, *et al.*, ZYP1 is required for obligate cross-over formation and cross-over interference in Arabidopsis. *Proc. Natl. Acad. Sci. U. S. A.* **118**, 1–11 (2021).
51. N. Vrielynck, *et al.*, SCEP1 and SCEP2 are two new components of the synaptonemal complex central element. *Nat. plants* **9**, 2016–2030 (2023).
52. L. Cromer, *et al.*, Rapid meiotic prophase chromosome movements in Arabidopsis thaliana are linked to essential reorganization at the nuclear envelope. *Nat. Commun.* **15**, 5964 (2024).
53. J. D. Hollister, *et al.*, Genetic Adaptation Associated with Genome-Doubling in Autotetraploid Arabidopsis arenosa. *PLoS Genet* **8** (2012).
54. K. M. Wright, *et al.*, Selection on Meiosis Genes in Diploid and Tetraploid Arabidopsis arenosa. *Mol Biol Evol* **32**, 944–955 (2014).
55. M. Bohutínská, *et al.*, De Novo Mutation and Rapid Protein (Co-)evolution during Meiotic Adaptation in Arabidopsis arenosa. *Mol. Biol. Evol.* **38**, 1980–1994 (2021).
56. A. De Muyt, *et al.*, A high throughput genetic screen identifies new early meiotic recombination functions in Arabidopsis thaliana. *PLoS Genet.* **5** (2009).
57. S. Tessé, *et al.*, Asy2/Mer2: an evolutionarily conserved mediator of meiotic recombination, pairing, and global chromosome compaction. *Genes Dev.* **31**, 1880–1893 (2017).
58. C. Lambing, *et al.*, Differentiated function and localisation of SPO11-1 and PRD3 on the chromosome axis during meiotic DSB formation in Arabidopsis thaliana. *PLoS Genet.* **18**, 1–27 (2022).
59. M. Grelon, D. Vezon, G. Gendrot, G. Pelletier, AtSPO11-1 is necessary for efficient meiotic recombination in plants. *EMBO J.* **20**, 589–600 (2001).
60. F. Couteau, *et al.*, Random chromosome segregation without meiotic arrest in both male and female meiocytes of a dmc1 mutant of Arabidopsis. *Plant Cell* **11**, 1623–34 (1999).
61. C. Oliver, J. L. Santos, M. Pradillo, On the role of some ARGONAUTE proteins in meiosis and DNA repair in Arabidopsis thaliana. *Front Plant Sci* **5**, 177 (2014).
62. J. B. Fernandes, *et al.*, FIGL1 and its novel partner FLIP form a conserved complex that regulates homologous recombination. *PLoS Genet.* **14**, e1007317 (2018).
63. W. Xu, *et al.*, SCFRMF mediates degradation of the meiosis-specific recombinase DMC1. *Nat. Commun.* **14**, 5044 (2023).
64. L. Chu, *et al.*, ASYNAPSIS3 has diverse dosage-dependent effects on meiotic crossover formation in Brassica napus. *Plant Cell* **36**, 3838–3856 (2024).
65. M. Leflon, *et al.*, Crossovers Get a Boost in Brassica Allotriploid and Allotetraploid Hybrids. *Plant Cell* **22**, 2253–2264 (2010).
66. A. Pecinka, W. Fang, M. Rehmsmeier, A. a Levy, O. Mittelsten Scheid, Polyploidization increases meiotic recombination frequency in Arabidopsis. *BMC Biol.* **9**, 24 (2011).
67. E. L. Stamper, *et al.*, Identification of DSB-1, a Protein Required for Initiation of Meiotic Recombination in Caenorhabditis elegans, Illuminates a Crossover Assurance Checkpoint. *PLoS Genet.* **9**, 1–18 (2013).

68. D. Thacker, N. Mohibullah, X. Zhu, S. Keeney, Homologue engagement controls meiotic DNA break number and distribution. *Nature* **510**, 241–6 (2014).
69. J. Westermann, T. Srikant, A. Gonzalo, H. S. Tan, K. Bomblies, Defective pollen tube tip growth induces neo-polyploid infertility. *Science* **383**, eadh0755 (2024).
70. S. Marburger, *et al.*, Interspecific introgression mediates adaptation to whole genome duplication. *Nat. Commun.* **10**, 5218 (2019).
71. C. Morgan, E. Wegel, Cytological Characterization of Arabidopsis arenosa Polyploids by SIM. *Methods Mol. Biol.* **2061**, 37–46 (2020).
72. J. C. Yen, F. J. Chang, S. Chang, A New Criterion for Automatic Multilevel Thresholding. *IEEE Trans. Image Process.* **4**, 370–378 (1995).
73. W. H. Tsai, Moment-preserving thresholding: a new approach. *Comput. Vision, Graph. Image Process.* **29**, 377–393 (1985).
74. H. Wickham, Elegant Graphics for Data Analysis. *ttps://ggplot2.tidyverse.org* (2016).
75. E. Brooks, Mollie, *et al.*, glmmTMB Balances Speed and Flexibility Among Packages for Zero-inflated Generalized Linear Mixed Modeling. *R J.* **9**, 378 (2017).
76. D. Lüdecke, M. Ben-Shachar, I. Patil, P. Waggoner, D. Makowski, performance: An R Package for Assessment, Comparison and Testing of Statistical Models. *J. Open Source Softw.* **6**, 3139 (2021).
77. F. Hartig, DHARMa: Residual Diagnostics for Hierarchical (Multi-Level / Mixed) Regression Models. *http://florianhartig.github.io/DHARMa/* (2022).
78. R. V. Lenth, emmeans: Estimated Marginal Means, aka Least-Squares Means. *https://rvlenth.github.io/emmeans/* (2024).
79. C. Lambing, *et al.*, Arabidopsis PCH2 Mediates Meiotic Chromosome Remodeling and Maturation of Crossovers. *PLoS Genet.* **11**, 1–27 (2015).
